# Address on the Pursuit of Professional Excellence

**Published:** 1845-09

**Authors:** John Allen


### ARTICLE IV.

*Address on the Pursuit of Professional Excellence.* Delivered before the American Society of Dental Surgeons, August 8th, 1845. By JOHN ALLEN, D. D. S.

*Mr. President, and Fellows of the*
*American Society of Dental Surgeons:*

HAVING assembled for the purpose of uniting our efforts in promoting the requisite knowledge of the dental science, and for inspiring a laudable zeal for the elevation of the art, by mutual and reciprocal interchange of opinions, and experience connected therewith, you will allow me to congratulate you, and the whole fraternity of our profession, upon the fact, that we are not obliged to hang our harps upon the willow, and yield obedience to infuriated zeal or blind fanaticism, but are permitted to bring to bear upon our favorite profession the highest grade of mental power, mechanical skill, and scientific research. The dark clouds of ignorance and obscurity, that have so long hung heavily over our craft, are now vanishing away, and the dawn of brighter days are smiling upon us, and already do we find ourselves reposing in the confidence of an intelligent and liberal community.

And it now becomes our duty so to preserve and so to demean ourselves in our vocation, as to justify the fond hope that ere long we shall be seated on the mountain's brow of science, possessing and enjoying a lofty pinnacle of professional fame.

A profound knowledge of anatomy, physiology, and pathology, is indispensable to the practical dentist; and he who is deficient in such knowledge, can never attain to that lofty summit of celebrity, to which the truly scientific mind delights to aspire.

Every day's experience teaches the importance of forming societies for the promotion of mutual good. The concentrated efforts of an association like this naturally lead the way to a development of all the attainments of our profession, giving each member an opportunity to profit by the skill and experience of all the others, whilst they serve to qualify the industrious operator, and to share the pleasures which advancement in the art must necessarily afford.

An association established for noble purposes cannot fail, if properly conducted, to contribute largely to the advancement of all its members. It is calculated to unite men of different states and different countries, and to cultivate friendly intercourse among those who might otherwise have remained at perpetual distance. That towering temple erected on Mount Moriah's top more than twenty-three hundred years ago, and on which no sound of axe, hammer, or other tool of iron, was heard, never could have been completed, without the united efforts of master builders; for it is as necessary to have union to support, as wisdom to contrive, in accomplishing and sustaining any noble enterprise. In all ages of the world, more good has been done by well regulated societies than possibly could have been done by persons acting in an individual capacity. We see in those barbarous countries, upon which the light of science has never dawned, and where civilization remains yet unknown, that societies are never formed, either for the purpose of improving the mind, or in any respect bettering the condition of man. Hence, we find that for the last four thousand years, the inhabitants of those regions have made no more improvements even in the construction of their own domicils, than have the beaver, the otter, or the stork of the forest.

How important then is an association like this of ours. Here the accomplished teacher of our art points out objects worthy the attention of its members, and directs their minds to the path that leads to fame. Here the young practitioner is inspired with a becoming zeal to do honor to the profession of his choice, to become a useful member of society, and to adorn the circle in which he moves. Although from an early period in the history of diseases incident to the human teeth and gums, very many able works have been written, and many scientific investigations and experiments have been made, by ingenious and pre-eminent gentlemen, who have been regarded as stars in the galaxy of the profession of dental surgery, yet more truly valuable improvements and discoveries have been made within the last twenty years, than had been previously brought to light from time immemorial; and your speaker, who now addresses you, would here remark, that he has recently added one more link to the long chain of improvements that have been made in connection with our profession. Which is that of restoring the form of the face to its original fullness, in cases where the cheeks have fallen in, in consequence of the loss of the teeth, absorption of the alveolar processes, wasting of the flesh or loss of the maxillary bones, which is done by means of gold or other attachments to the teeth in such a manner as to give just that form to the face that may be desired, thereby changing the thin lank and deeply furrowed contour of the cheeks to a smooth, plump, and beautiful form.

Having tested fully the practicability of this improvement, I most respectfully submit it to the consideration of this association. Though the task of acquiring a thorough knowledge of the dental art may be irksome to the inexperienced, yet perseverance and close application will remove each difficulty as it occurs; and at every step he advances, new and pleasing discoveries open to his view, and additional pleasures will attend all his researches, and impress more and more strongly on the mind the importance of attaining a high degree of eminence in his profession. Although he may meet with obstructions on his way to the portals of usefulness and of fame, and although he may sometimes meet with momentary disappointment, yet he must not suffer his timidity to deter or discourage him, but he must press forward until he becomes more than conqueror, over all the obstacles that oppose him. And yet, he must not expect to be wrapt in clouds of glory, nor suffer his senses to be overwhelmed by a fancy that peals of thundering applause are especially held in reserve to gratify his vanity, but he must pass on to the highest attainments in his power, and the highest honors that he may have merited will be conferred upon him. By a proper discharge of his duties in all things, he may enjoy a reputation more honorable than the diadems of kings or the pearls of princes can confer.

The science of dentistry is a fruitful one; and although the operator should be completely master of his business, and should be capable of illustrating and elucidating its principles in an interesting and forcible manner, yet he is not confined to any set forms or rules of action, but in his modus operandi must be governed by the circumstances surrounding the cases to which his attention may be directed.

I have spoken of perseverance as being essential to the surgeon dentist, for without it, nothing great, noble, or extensively useful has ever been accomplished. It was this feature in the character of Tubal Cain that enabled him to become the first successful artificer in brass and iron, and to lay the foundations of almost all the useful mechanic arts. It was perseverance, coupled with energy and enterprise, that enabled righteous Noah to construct that ark of safety which was permitted to float on the rolling waves of a universal deluge; and thereby preserved him, his family, and all that he took with him, from the destructive waters which covered the whole surface of the earth. With what perseverance did the Chaldean shepherds, with whom the science of astronomy originated, pursue their researches and observations, in reference to the positions, relative distances, and motions of the heavenly bodies. And surely the Egyptians, who discovered the fundamental principles of geometry, and reduced it to a science, the importance of which has been realized by all the civilized nations of the earth, could never have succeeded without the most unflinching perseverance, and indefatigable exertion. So with the five beautiful orders of architecture, which have remained for centuries without any improvement; and which have contributed so much to the comfort and convenience of man, and at the same time serving as ornaments throughout all the enlightened portions of the globe.

Surely such perfection in art could never have been attained without the most untiring energy and patient investigation. The same may be said of all the other arts and sciences, as well as of all the other discoveries that have been made, and that have a tendency to improve the condition or promote the happiness of man. What but perseverance, combined with boldness and energy, enabled Charles the Seventh to break down the whole feudal system; the prevalence of which for the space of four hundred years, filled Europe with more atrocities than was ever known during any other period in the annals of that country. He not only checked the licentiousness of the barons, but he crushed the power of the nobles, and reduced them to order and obedience. The objects at which he aimed were to establish a corrective of disposition, and to support the weak, protect the oppressed, to refine the rude, to avenge wrongs, to maintain the rights of all, and especially to defend the purity of the lovelier sex. But for the spirit of enterprise and perseverance, this happy and highly cultivated country, through which we can now travel with such velocity and ease, and in which there are so many magnificent temples erected and dedicated to the Most High, and in which are so many stately mansions constructed for the safety and comfort of man, would to this day have been a dark and howling wilderness, inhabited only by the untamed savage and the wild beasts of the forest. But for that spirit, the thousands of ships, which now ride sternly over the bounding billows of oceans, seas, and lakes, would never have been put in motion or even constructed. Nor would the government of this *our* native land, have been wrested from the power of despotism, but the people would to this day have been the subjects of monarchy, or reduced to a state of barbarism, anarchy and confusion. And for this spirit are we indebted for almost all the joys that sweeten life, as well as for that lofty position which distinguishes man from the lower orders of creation.

I have thus strongly, and with anxious solicitude, urged the necessity of deep and persevering research in the science of our profession, because I feel sure that hitherto much less attention has been bestowed upon it than its importance demands.

The practical and judicious dentist will often have his attention directed to diseases which are intimately connected with his profession, and yet not confined exclusively to the teeth and gums, such as diseased antrum; bony tumors, which are sometimes attached to the bones of the face; deranged arch of the mouth, contractions of the cheeks, diseased or absent palate, various forms of exostosis, hæmorrhage, &c., &c.; all of which go to show the importance of a thorough knowledge of the physiology, pathology and anatomy, not only of the mouth and face, but all the other parts connected therewith.

The dentist should not only qualify himself for operating successfully, but should also cultivate a gentlemanly deportment, preserve a reasonable degree of dignity, and manifest a becoming sympathy for his suffering patients. He should exhibit no signs of fear or alarm in their presence, but be sure that he is adequate to the task imposed upon him, and then proceed with boldness and precision to the execution of whatever is proper for him to do. By strictly observing these rules, he will generally avoid material error, and suffer but little from disappointment. In all his professional duties, it will be incumbent upon him to be governed by that high sense of honor, delicate sensibility, and friendly feeling, that are calculated to secure the implicit confidence of all who may have entertained a favorable opinion of his skill and integrity. By securing the entire confidence of his patients, the dentist is enabled to hold in check that timidity which is often much to be dreaded, always unpleasant, and which frequently serves to defeat the designs of the operator. He should always remember never to express the least doubt, in the hearing of his patient, as to the result of any practical operation which he is about to perform.

It seems to have been ordained, that man should be the channel through which good should be conveyed to his fellow-man, hence the necessity of organised societies like this; and here let me remark, that every member of this association ought to consider himself bound by honor to preserve unsullied his own reputation, and to his utmost ability, that of each member of the society. If he wishes to secure for himself an honorable reputation, he must first possess the qualifications already described; and, secondly, he must ever cherish the remembrance of that connecting link between divinity and humanity—I mean that noblest of virtues, which is *charity*, and which is a perpetual current of good, extended to all mankind, but more especially towards the children of sorrow, want and pain. Although he is not required to extend his benevolence so far as to adorn or decorate those who are unable to remunerate him, yet no pecuniary consideration should ever induce him to withhold his aid from suffering humanity; but his professional duties, as connected with the healing art, should excite and awaken all the noble affections of the human heart, and call into exercise all those principles and actions that are calculated to mitigate the sorrows, assuage the griefs, and relieve the afflictions of his fellow mortals, at least as far as he can consistently, for there are many who have been driven by the cruel winds of adversity from the fairest prospects of prosperity and plenty into the depths of poverty, want and despair, and over whom the clouds of misfortune have long seemed to hold dominion, and whose dreams of delight are forgotten, and who, at the same time, are writhing with pains that baffle description; and shall the avarice of any one of our profession lift her supercilious brow above such scenes, or behold them with indifference, and prefer to be influenced by the cringing flattery of knaves, who only aim to touch his vanity or tickle his folly? And yet such scenes as are above described are frequently witnessed by those whose attention is exclusively devoted to the relief of the afflicted. What a noble example of this spirit was presented to us yesterday by Dr. Townsand, in the character of that great and good man, Dr. Hudson. Such examples are worthy of all commendation. Let that virtue which charity inculcates, that shines refulgent on the mind, and that is as luminous as the meridian sun, and that virtue which enlivens the heart and converts coldness and indifference into warm sympathy and cordial affection, be our polar star during our whole pilgrimage through this uncertain world of care, of joy, and of sorrow.

The science of dental surgery is yet far from being perfectly known or understood at the present day; but many of its elementary truths, and fundamental principles, are yet floating on the broad ocean of human knowledge, and it is ardently hoped that some master minds of this association will collect them together, and rear upon them a scientific structure not inferior to any ever formed in the wisdom of man.

Theories will sometimes be urged, that will, when fairly tested, prove vain and useless. Other theories will be presented and rejected by many, because they appear not to be in conformity with some pre-conceived notions of truth; but let every hypothesis, not known to be palpably opposed to truth, lead to a close investigation; and he who reasons well, investigates thoroughly, and employs his mind and his hands industriously, may feel an assurance that he, in his profession, is marching forward to certain conquest, and that the lofty objects of his ambition will be finally realized. I think there is now very much to encourage expectation, and to give increased activity to the enterprising members of this association, for we inhabit a country where the human mind can avail itself of its full strength, and where many of the useful arts have been invented, and many more improved —where commerce and intercourse are facilitated by seas, lakes, rivers, canals, railways and turnpike roads, and where intelligence is speedily conveyed. We live, too, in a country that may be regarded as an immense treasury of nature, producing most of the mines, minerals, and other materials necessary for the prosecution of our art, together with all the other productions of earth necessary for the support of man. Here are presented to us the most magnificent spectacles of attraction, and the most lofty munificence that the world ever knew, and which are calculated to impregnate the mind with enterprise, and the highest possible sense of love and of gratitude. And here our wants are supplied by a boundless plenty; here are exhilirating benedictions bestowed upon us, to cheer our hearts, employ our thoughts, improve our intellectual powers, and finally lead us forward to greatness, to goodness, and to fame.

And shall we, with all these advantages, permit ourselves to become drones in the hive of nature, or, through indolence or apathy, allow our profession to suffer disgrace, or ourselves to relax into a state of insignificance, wantonness and folly? The object of our pursuit is magnanimous, it is noble—it is to relieve and comfort the afflicted, to inculcate social affections, sweeten temporal enjoyments, remove disquietude, and strengthen the common bands of harmony and of interest. How cautious then ought we to be in the admission of members into our association. Although we want no specimens of nobility amongst us, yet we wish to see bright stars in our ranks—men distinguished for their high attainments, for their noble qualities of soul, and for every virtue which can give dignity to the human character; in short we want such men as nature has made noble, and such as are disposed to improve their stock of knowledge by every opportunity afforded them. We want such men as can reciprocate the sentiments and emotions of their brethren, and mingle in the enjoyments which occasions like this furnish. We want men of clear heads and pure hearts, of real worth, unostentatious, endeavoring to appear just what they really are, entirely above the littleness of wishing to render themselves conspicuous without merit. Let our association be composed of such men, and all the standard bearers of scepticism cannot shake our constancy or defeat our aims.

Finally, brethren, let our abiding motto be, onward in the march of improvement—onward in the march of science, invention, skill, perseverance, enterprise, and above all, the faithful discharge of all our solemn obligations.

### ARTICLE V.

*Dissertation on the State of the Dental Profession, and Dental Empiricism.* Delivered before the American Society of Dental Surgeons, at their Sixth Annual Meeting, held in the City of New York, August, 1845. By JAMES TAYLOR, D. D. S.

*Mr. President, and Gentlemen*

*of the American Society of Dental Surgeons:*

